# The Potential Mechanisms of the Neuroprotective Actions of Oil Palm Phenolics: Implications for Neurodegenerative Diseases

**DOI:** 10.3390/molecules25215159

**Published:** 2020-11-05

**Authors:** Nurul ‘Izzah Ibrahim, Nur Balqis Muhammad Ismail Tadj, Md. Moklesur Rahman Sarker, Isa Naina Mohamed

**Affiliations:** 1 Pharmacoepidemiology and Drug Safety Unit, Department of Pharmacology, Faculty of Medicine, Universiti Kebangsaan Malaysia Medical Centre, Jalan Yaacob Latif, Bandar Tun Razak, Cheras, Kuala Lumpur 56000, Malaysia; nurulizzah88@gmail.com (N.I.‘I.); balqistadj@gmail.com (N.B.M.I.T.); 2 Department of Pharmacy, State University of Bangladesh, 77 Satmasjid Road, Dhanmondi, Dhaka 1205, Bangladesh; moklesur2002@yahoo.com

**Keywords:** oil palm phenolics, oxidative stress, antioxidant, anti-inflammatory, neuroprotection

## Abstract

Neurodegenerative diseases (ND) can be characterized by degradation and subsequent loss of neurons. ND has been identified as the leading cause of disability-adjusted life years (DALYs) worldwide and is associated with various risk factors such as ageing, certain genetic polymorphisms, inflammation, immune and metabolic conditions that may induce elevated reactive oxygen species (ROS) release and subsequent oxidative stress. Presently, no specific cure or prevention is available for ND patients; the symptoms can be only alleviated via drug treatment or surgery. The existing pharmacological treatments are only available for partial treatment of the symptoms. A natural product known as oil palm phenolics (OPP), which is high in antioxidant, could become a potential supplementary antioxidant for neurodegenerative health. OPP is a water-soluble extract from palm fruit that demonstrated medicinal properties including anti-tumor, anti-diabetic and neuroprotective effects. In this review, OPP was proposed for its neuroprotective effects via several mechanisms including antioxidant and anti-inflammatory properties. Besides, OPP has been found to modulate the genes involved in neurotrophic activity. The evidence and proposed mechanism of OPP on the neuroprotective health may provide a comprehensive natural medicine approach to alleviate the symptoms of neurodegenerative diseases.

## 1. Introduction

Neurodegenerative diseases (ND) is a heterogeneous group of disorders that can be characterized by degradation and subsequent loss of neurons [1]. The degradation of neurons in the human brain may lead to functional or sensory deterioration of the patients over time [2]. ND, which include Alzheimer’s disease (AD), Parkinson’s disease (PD) and Huntington’s disease (HD) may contribute to major health and financial issues to health service organization [3]. ND has been reported as the leading cause of disability-adjusted life years (DALYs) and the second leading cause of death worldwide [4]. Several risk factors have been identified for ND including ageing and certain genetic polymorphisms. Besides, oxidative stress, inflammation, vitamin deficiencies, immune and metabolic conditions as well as chemical exposure have been linked to ND [5]. Among all causes, ageing is the primary risk factor for most ND [3].

During ageing, irreversible changes may occur such as the loss of irreplaceable cells in brain, heart and skeletal muscles. Specifically, neurons in the brain may shrink and disappear, whereas alterations may take place in neuronal synapses and networks. Due to the neuron loss particularly in hypothalamus region of the brain, certain physiological changes such as altered metabolism and circadian rhythm, which might be closely related to the mental and emotional aberrations in the elder group, may occur [6]. Most neurodegenerative diseases commonly occur in the older generation (late-onset) [7]. Ageing may also result in decreased catecholamine sensitivity [8], whereby the catecholamines play many roles in important brain functions such as attention, memory, cognition and emotion [9]. Therefore, the decreased catecholamine sensitivity may contribute to many repercussions [9,10]. Meanwhile, at the cellular level, ageing has been linked to accumulation of oxidative stress, reduction of mitochondrial function, incidence of telomere erosion, impairment in DNA repairing process and reduction in tissue regeneration [11].

Apart from that, neurodegenerative diseases have been associated with aggregation of cytoskeletal protein, which then developed to inclusions in neurons and/or glial cells [12]. The accumulation of the abnormal aggregated proteins can in turn trigger inflammatory response in the brain, which may induce elevated reactive oxygen species (ROS) release and subsequent oxidative stress [13]. Oxidative stress can be defined as a condition of imbalance between the formation of ROS and capacity of cellular antioxidant due to enhanced ROS generation and/or dysfunction of the antioxidant system [12]. Elevated ROS level may worsen disease progression via oxidative damage and interaction with mitochondria. For mitochondria, the generation of ROS known as NADPH oxidase (Nox) and xanthine oxidase (XO) remains at relatively low level by the activity of endogenous antioxidants [14]. However, the level of ROS in the mitochondria can be disturbed by neural inflammation or abnormal mitochondrial function. Thus, the regulation of the cellular ROS levels should be taken up for providing a potential preventive and treatment care to obstruct neurodegeneration and alleviate associated symptoms [15,16]. Currently, no cure or prevention is available for ND patients; the symptoms can only be alleviated via drug treatment or surgery. Moreover, the pharmacological treatments are only available for partial treatment of the symptoms [17]. Previous clinical studies have shown that neurodegenerations can be alleviated upon proper intake of natural or supplementary antioxidants [18].

A natural product known as oil palm phenolics (OPP), which is high in antioxidants, could become a potential supplementary antioxidant for neurodegenerative health. OPP is a water-soluble extract from palm fruit (*Elaies guineensis*) and is the product filtrate recovered from aqueous waste stream during palm milling process. Globally, there are approximately 85 million tonnes of vegetation liquor are discarded annually into the aqueous waste stream. The development of a novel recovery procedure to isolate phenolic compounds from the aqueous waste stream contributes to the OPP production. With the growing evidence that phenolics originated from plants may contribute to health benefits, the assessment of OPP for its potential bioactivities has been actively explored. The main active ingredients of OPP have been identified, which include caffeoylshikimic acid (CFA), *p*-hydroxybenzoic acid (PHBA), protocatechuic acid (PCA) and hydroxytyrosol [19]. These active ingredients of OPP have been also associated with various positive health effects [20]. Under appropriate conditions, hydrolysis of CFA can produce caffeic acid (CA) and shikimic acid (SA) [21]. SA is an important component for oseltamivir phosphate (Tamiflu^®^), a drug used for prophylaxis and treatment of several influenza viruses such as swine-origin influenza virus H1N1, types A and B seasonal influenza virus and avian influenza virus H5N1 [21,22]. The ability of CFA to be hydrolysed into SA has gained much attention from researchers as there is a significant growing demand for Tamiflu and supplies SA from the primary source of SA, Chinese herbal star anise (*Illicium verum*) are limited [23]. Other pharmacological potentials of SA include neuroprotective, anti-inflammation, anti-hyperalgesia, anti-thrombogenic and ability to improve colon damage [20]. For PHBA, its esters such as methyl-, ethyl-, propyl or butylparaben have been proven as potent antimicrobial agents. Therefore, the esters of PHBA are extensively used as preserving agents in food, cosmetics, and pharmaceutical industries [24]. Other phenolic compounds such as quercetin and delphinidin have also been identified to possess antimicrobial potential due to ability to reduce biofilm formation of the *Pseudomonas aeruginosa* PAO1 [25,26]. PHBA has been discovered to possess anti-hyperglycemic activity as tested on normal and diabetic-induced rat models [27,28]. Meanwhile, another component of OPP, PCA has demonstrated anti-hyperglycemic activity in a series of pre-clinical studies [29,30,31]. Apart from that, PCA has also shown anti-inflammatory, anti-hyperlipidemia and anti-atherogenic [32,33]. Hydroxytyrosol, which is also present in olive oil, has shown anticancer effects in several in vitro studies [34,35,36]. Besides, hydroxytyrosol has also shown its potential for the prevention and treatment of cardiovascular and neurodegenerative diseases [37,38,39]. These studies have demonstrated that the individual components of OPP have unique pharmacological potential in preventing and treating various diseases, which might also contribute to synergistic effect when all components exist in the OPP. To date, previous studies have shown medicinal properties of OPP including anti-tumour, anti-diabetic, atheroprotective [40,41] and neuroprotective effects [42,43]. Specifically, water-soluble phenolic compounds present in other natural products are beneficial for neuroprotection. For example, water-soluble polysaccharides extracted from Ganoderma lucidum (oriental fungus) have shown their protection against cerebral ischemic injury by downregulating caspase-3 activation and modulating the Bcl-2/Bax ratio, which in turn may inhibit apoptosis [44]. Apart from that, phenolics extracted from Brazilian green propolis have also shown neuroprotective effect against oxygen-glucose deprivation induced retinal ganglion cell line [45].

For OPP, its neuroprotective effects could be proposed by several mechanisms including antioxidant [43] and anti-inflammatory properties [42,46]. In addition, the neuroprotective effects of OPP have been associated with its ability to upregulate the genes involved in neurotrophic activity [42]. This information is important to be implicated for future development of a comprehensive natural medicine approach in alleviating the symptoms of neurodegenerative diseases.

## 2. Effects of Oil Palm Phenolics on Nervous System

The direct effects of OPP in relation to neurons have been identified (Table 1). In a study by Leow et al. [42], a weekly assessment for 6 weeks with water maze and rotarod test has been performed to OPP-supplemented BALB-c mice to assess the effects on cognitive and motor functions. OPP was provided as a drinking fluid at 1500 ppm GAE antioxidant content to the mice. It was shown that the trend in latency and mean distance to platform in a water maze test was decreased compared to that of the control group, which had constant values throughout the period. The OPP-supplemented group displayed slightly increased mean velocity at the initial time of the test compared to the control, whereas the differences were insignificant towards the end of the study, which revealed improvement in spatial learning instead of swimming speed in the OPP-treated mice [42]. The water maze was a test of spatial learning for rodents based on the principle that the animals have evolved an optimal strategy to explore the open swimming arena and escape from water with minimum effort by swimming the shortest distance possible [47]. In the same study, rotarod test has been also performed to determine the mice balancing abilities. The OPP-treated group mice showed increased trend of average time, average distance travelled and average stopping speed in rotarod test before they fell off the rotating drums compared to the controls. This observation may suggest that OPP supplementation may have improved balance and motor coordination [42]. In the study, the modulation of brain gene expression by OPP was also investigated for determining the molecular mechanisms involved. OPP had upregulated genes involved in neurotrophic activity such as Arc, Cast or D14Ertd171e, Gria3, Kcnb1, Kcnab1, Homer1, Dlgap2, Dlgh4, Sv2b, Stx1a, Gucy1b3, Ncald, Bzrap1 and Pclo [42]. These upregulated genes were all under the regulation of brain-derived neurotrophic factor (Bdnf) that plays an important role in regulating neuronal survival, differentiation, growth and apoptosis [48]. OPP has been also found to downregulate genes involved in inflammation such as Spp1, Saa3 and Apod [42]. The improvement of cognitive and motor functions shown by the water maze and rotarod tests might be associated with the ability of OPP to upregulate the genes involved in neurotrophic activity and downregulate the genes involved in inflammation.

A series of experimental studies has been conducted to investigate the effects of OPP in preventing or treating brain amyloidosis [46], a condition where various proteins are deposited and associated with different neurodegenerative diseases [49]. An in vitro study subjected to monitor amyloid β (Aβ) protein fibril formation using OPP at 300 µl/10 mL on Thioflavin T (ThT) binding assay has been performed. It was shown that the ThT fluorescence intensity of OPP-supplemented assay was decreased when compared to the control [46]. In another in vitro study by Weinberg et al., OPP had inhibited the aggregation of Aβ into oligomers and significantly reduced the cytotoxicity of aggregating neuropeptides in yeast genetically engineered to overexpress these peptides. This inhibition and cytotoxicity reduction activities may suggest the potential of OPP in reducing neuroinflammation and neuronal death, therefore able to prevent or treat Alzheimer’s disease [50]. In the brain, the accumulation of Aβ protein is assumed as an early toxic event in the development of Alzheimer’s disease, which is the most common form of dementia linked with plaques and tangles in the brain [51]. In ThT fluorescence method, the ThT fluoresces only bound to the protein fibrils [52], indicating that the OPP may contribute to the reduction of Aβ plaques and therefore halt the pathogenesis of AD. Meanwhile, via western blot analysis, OPP have shown a reduction in the protein expression levels of amyloid precursor protein (APP), cyclo oxygenase 2 (COX-2) and poly (ADP-ribose) polymerase (PARP) in mutant and wild type (WT) cells. APP, COX-2 and PARP are well-known inflammatory biomarkers for Alzheimer’s disease. It was also reported that OPP at concentration of 300 μL/10 mL have lowered the APP levels in FAD cells to a level expressed in the WT control sample, reflecting the ability of OPP to reduce the APP levels on individuals without Alzheimer’s disease. Although the protein expression data has demonstrated that OPP had the right direction upon decreasing certain inflammatory biomarkers associated with AD, a study using RT-PCR has been conducted to determine the effects of OPP at the mRNA level. It was found that at the mRNA level, APP in FAD cells was not significantly different between the control and OPP-containing samples, which might indicate that the OPP do not affect cells at the mRNA level. In contrast, the mRNA level of APP expression in WT cells showed a statistically significant fold difference, when comparing the WT control and WT 150 to WT 300 and WT 600 μL/10 mL samples. Meanwhile, for COX-2 mRNA levels in FAD cells, concentration of 150 μL/10 mL was the only sample to show a significant difference, which might suggest contamination to the sample. Meanwhile, the fold change for COX-2 mRNA expression in WT cells was similar to APP. This may indicate that OPP may affect non-AD when extrapolated to humans.

An in vivo study using OPP at 5% concentration given in purified diet has been performed to determine the effects of OPP on beta amyloid deposition in cholesterol-induced rat model of Alzheimer disease [53]. In the study, the rats were fed with cholesterol-enriched diet that can impair cholinergic system leading to memory deficits. This was evidenced by the rats of high-cholesterol group demonstrating impaired learning and long-term memory in the Morris water maze experiment [53]. Hypercholesterolemia can also contribute to the formation of extracellular beta amyloid plaques in the brain hippocampus region that resembles an AD-like pathology causing neuronal loss [54]. The OPP supplemented to the cholesterol-induced rats showed better learning and cognition in Morris water maze test while demonstrating few traces of amyloid deposition compared to high cholesterol group rats for the histological findings. These results may indicate that OPP has provided neuronal protection and decreased the beta amyloid plaque deposition in the hippocampus region of the rat model of Alzheimer disease.

Besides, an in vivo study that also supplemented 5% OPP embedded in high-cholesterol purified diets to the aged rats for 23 weeks has been performed [43]. This study aimed to investigate the behavioral change and pathology upon the OPP supplementation in the atherogenic diet to induce memory deficit characteristics, which resembling Alzheimer’s disease. In the study, investigation for improvement in the animals’ spatial learning ability for the feeding period was performed by Morris Water Maze test by subtracting the escape latency (EL) at M4 from M1.

It was shown that the OPP-fed rats had a significant improvement in EL (*p* < 0.05) compared to control and high-cholesterol groups. It was also found that OPP-treated group preserved more healthy neurons in H&E staining while demonstrating lower density of plaque deposition in the hippocampal CA-1 and dentate gyrus (DG) areas when compared to high-cholesterol fed diet rats [43]. This finding implicates that OPP may have improved the condition of hippocampus, which is the brain region that regulates memory and learning ability and particularly vulnerable to neuronal dysfunction in the earliest stage of AD [57]. The OPP also demonstrated significantly lower β-amyloid 42 level when compared to control and high-cholesterol diet groups [43]. β-amyloid 42 is the major constituent of senile amyloid plaque in the brain affected with AD [58]. OPP-fed group also had significantly lower plasma, liver and brain MDA level compared to high-cholesterol fed group [43]. This suggests that OPP can lower the toxic aldehyde level in blood, as well as reducing the lipid peroxidation in the liver tissues and brain oxidative level. Measurement of ketone 3-hydroxibutyrate (3-HOB) in rat urine samples showed that OPP-fed group had significantly lower 3-HOB level compared to high cholesterol group [43]. Ketone formation is associated with radical termination of fatty acid formation [59]. However, in this study, OPP showed insignificant difference in the level of inflammatory marker, IL-6, when compared to high cholesterol group [43]. In the same study, gene expressions of APP, BACE-1 and Apo E associated with plaque formation in the brain were also measured. For OPP-fed group, the expressions of APP and BACE-1 genes were significantly lower compared to high-cholesterol diet group, which implicate the ability of OPP to down-regulate these enzymes and inhibit the amyloid pathway against the plaque formation direction. OPP had also significantly lowered the ApoE gene expression compared to high cholesterol group [43]. ApoE gene is responsible for encoding the ApoE lipoprotein, which serves as a reservoir employed for cholesterol transport between neuronal and non-neuronal cells [60].

Another in vitro study had supplemented 800 µg/mL OPP for 12 h to U87 MG glioma cell line [55]. In the study, quantitative PCR and microarray studies were performed to determine the possibility of antioxidant gene to be regulated by OPP. It was shown that OPP had upregulated Phase II antioxidant genes namely PRDX2, HMOX1, XPA, GPX1, HSPB1, MAPK8, SOD1, PRdx1, GSTM4 and HIF1A ranging from 1.4x fold to 2.8x fold change. For microarray studies, mild up-regulation of glutathione synthase (GSS) and glutamate cysteine ligase, catalytic subunit (GCLC), were demonstrated with 1.4x and 1.2x fold change, respectively. Additionally, OPP treatment had increased the GSH/GSSG ratio of astrocytes that induce glutathione metabolism and subsequently affect the redox status of the cell. The effects of OPP on the protein expression of antioxidant enzyme (heme oxygenase 1) and key transcription (nuclear factor erythroid 2 related factor 2) were also described. In response to OPP at a concentration of 100 µg/mL up to 24 h, both HO-1 and nrf2 protein expressions have shown activation and significantly increased the expression in a time-dependently manner.

Another in vitro study has been conducted to determine the effects of OPP on liposaccharide (LPS) activated BV-2 cell line [55], which is a well-known in vitro system for studying the mechanism of neuron damage in activated microglia [61]. OPP was added at different concentration ranges from 50 to 800 µg/mL to the LPS-activated BV-2 cell line. In dose-dependent manner, the addition of OPP to the activated cell line demonstrated reduction in the protein levels of inflammatory enzymes such as inducible nitric oxide synthase (iNOS), cyclooxygenase-2 (COX-2) and prostaglandin-2. Additionally, OPP treatment had also significantly reduced the protein levels of a transcription factor, NF-κB in LPS-activated cells. These results may suggest that OPP are able to reduce the inflammatory responses in activated microglial cell line and potentially contribute to modulate inflammatory conditions in the nervous system [55].

An in vitro study using IL-1β-activated normal human astrocytes has been performed by Weinberg et al. 2018 [56]. In the study, OPP were added at concentration ranging from to 0, 0.9, 1.8, 3.6 mg/mL. The activation by IL-1β caused the astrocytes to proliferate and release various pro-inflammatory cytokines and chemokines such as TNFα, RANTES (CCL5), IP-10 (CXCL10) besides expressing specific cell surface biomarkers such as the intercellular adhesion molecule (ICAM), vascular cellular adhesion molecule (VCAM) and the neuronal cellular adhesion molecule (NCAM). In a dose-dependent and time-dependent manner, OPP had shown reduction in the level of TNFα, RANTES and IP-10. Moreover, OPP had also demonstrated significant reduction in ROS production by IL-1β-activated astrocytes. Additionally, OPP caused reduction in the expression of ICAM and VCAM, both in activated and non-activated human astrocytes [56].

## 3. Mechanism of Action of OPP in Neurodegenerative Diseases

### 3.1. Antioxidant Properties

Free radicals can be described as molecules with unpaired electron in their outer orbit. In a normal controlled physiological process, free radicals have a very important role in many biochemical activities such as signal transduction, gene transcription and regulation of soluble guanylate cyclase activity [15,62]. For example, Nitric oxide (NO) is a vital signalling molecule for the brain in modulating neuronal excitability, synaptic plasticity neurotransmitter release, as well as regulating rhythmic activity and neurovascular coupling [63,64,65]. Through numerous physiological and biochemical processes, the human body may produce oxygen free radicals and other reactive oxygen species as by products [15]. Oxygen-related free radicals (superoxide and hydroxyl radicals) and reactive species (hydrogen peroxide, nitric oxide, peroxynitrile and hypochlorous acid), are produced in the body primarily as a result of aerobic metabolism [66]. However, the overproduction of free radicals is associated with oxidative stress to biomolecules in the body including lipids, proteins and DNA, which may subsequently contribute to the development of chronic diseases such as atherosclerosis, cancer and other degenerative diseases in humans [15,67]. Generally, the human body has integrated antioxidant systems, which include enzymatic (superoxide dismutase, catalase and glutathione peroxidase) and nonenzymatic (coenzyme Q10, glutathione, uric acid, lipoic acid and bilirubin) antioxidants that are usually effective to counteract oxidative stress from the overproduced free radicals [68]. Factors contributing inadequate antioxidant defence of the brain include high rate of dioxygen utilisation, high content of unsaturated lipids [69], low level of glutathione, moderate activity of antioxidant enzymes (catalase, superoxide dismutases and glutathione peroxidase), high concentration of transition metals and ascorbic acid as well as the presence of neurotransmitters (which auto-oxidized to generate ROS). All of these factors overall contribute continuous production of ROS [70]. Prolonged oxidative stress to the CNS will prompt irreversible oxidative injury to the neuron macromolecules leading to neuronal cell death [71].

Plant phenolics have been well-known for their antioxidant properties due to the high redox potential that allows them to act as reducing agents, hydrogen donors, singlet oxygen quenchers and metal chelators. In neurons, antioxidants have the potential to prevent and treat neuronal disorders concerning the oxidative stress [7,72]. OPP have demonstrated their intrinsic antioxidant activity via two different assays: Trolox Equivalent Antioxidant Activity (TEAC) and Oxygen Radical Absorbance Capacity (ORAC). For TEAC, OPP demonstrated the ability to directly scavenge electrons in a dose-dependently manner. Meanwhile for the ORAC test, OPP have prevented the oxidation of fluorescein in a dose dependently manner [55]. Other than that, OPP have shown a significant scavenging activity with a half-life of less than 30 s at all concentrations tested ranging from 100 to 300 mg/L GAE [19]. The scavenging ability of OPP can be observed even at the lowest concentration used (100 mg/L GAE) in which more than 75% of DPPH were scavenged. The potent antioxidant activity of OPP is mainly contributed by the ability for free radicals scavenging and hydrogen atoms donation. In addition, the antioxidant property and free radical scavenging have been associated with hydroxylation degree of phenolic compound in which high hydroxylation degree will contribute to the high scavenging and antioxidant property [73]. To date, caffeoylshikimic acid, which is one of the individual components of OPP possesses four hydroxyl (OH^-^) groups that may be responsible for the potent antioxidant activity of OPP. Moreover, other phenolic acid contents in OPP such as protocatechuic acid and *p*-hydroxybenzoic acid may also contribute to the antioxidant property, which might act synergistically.

Oxidative stress (OS) is commonly considered as the initial point for the onset of several aging and chronic diseases, which undoubtedly plays a major role in the development of degenerative disorders [74]. OS in neuronal microenvironment might contribute to oxidation of lipid, proteins and DNA as well as the generation of many byproducts namely peroxides, alcohols, aldehydes, ketones and cholesterol oxide [75,76,77]. Most of the oxidative products are toxic to blood lymphocyte and macrophages, paralysing the in vivo defense system. In a study conducted by Wu 2017 [78], brain malondialdehyde (MDA) level was measured in atherogenic diet induced rat model of Alzheimer’s disease supplemented with OPP for five months. In the study, the OPP supplemented group showed that plasma, liver and brain MDA levels were significantly lower compared to those of high-cholesterol control group [78]. This indicates that OPP can reduce the lipid peroxidation in the liver tissues and able to lower the toxic aldehyde level in the blood and brain. MDA is a lipid peroxidation end-product and is the most popular indicator of oxidative damage to cells and tissues [79]. Moreover, OPP had also shown their ability to reduce ketone, a by-product of oxidative stress in hypercholestrolemic-induced rats. This implicates that OPP is able to reduce the effects of oxidative stress in an in vivo system [43]. In addition, OPP has scavenged the increased ROS in the L-NAME induced rat model, which involved the inhibition of endogenous nitric oxide production [40]. The ROS scavenging activity by OPP may reduce oxidative stress that might favor the development of neurodegenerative diseases.

**Figure 1:**
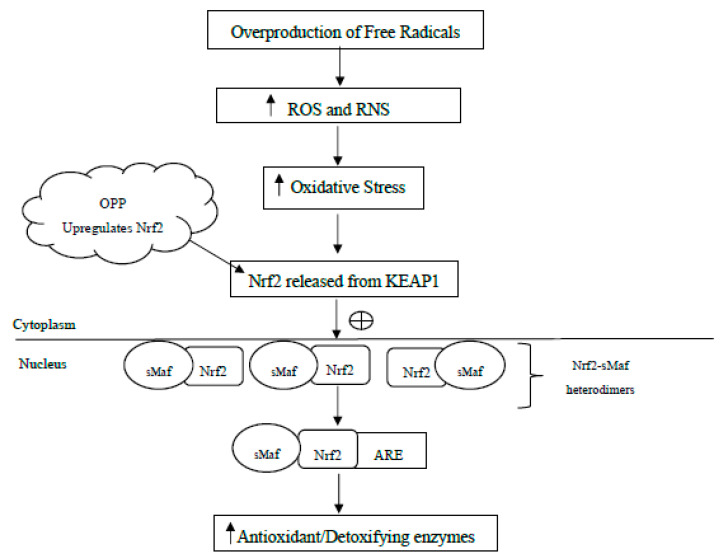
Keap-Nrf2-ARE pathway. A proposed mechanism for the antioxidant property of OPP. ⊕: activate or stimulate; RNS: reactive nitrogen species; ROS: reactive oxygen species; Nrf2: nuclear factor erythroid 2-related factor 2; KEAP1: Kelch-like ECH-associated protein 1; sMaf: small Maf proteins; ARE: antioxidant response element.

Sundaresan (2013) [55] has considered that the antioxidant property of OPP might be associated with Keap-Nrf2-ARE pathway (Figure 1). The study concluded this by considering the coordinated expression of several antioxidant response and phase II antioxidant genes by OPP. It was shown that OPP had upregulated phase II antioxidant enzymes, especially heme oxygenase 1 (HO-1) mRNA and activated Nrf2 protein expression on U87 MG cells [55]. HO-1 enzymes are responsible for electrophilic, xenobiotic material, conjugating glutathione or other moieties to the molecules to mark it for export from the cell. The up-regulation of HO-1 can be described as a crucial mechanism of cell adaptation to neuronal damage and neurodegeneration. Heme oxygenase may degrade heme groups to carbon monoxide (CO), free ferrous iron (Fe^2+^) and biliverdin. With the action of biliverdin reductase, biliverdin can be converted into the antioxidant bilirubin, that is able to scavenge free radical molecules [80,81]. Therefore, the increased expression of HO-1 in brain implicates a prospective defensive mechanism for neurodegenerative diseases. Other natural compounds with neuroprotective effects such as curcumin, carnosol and rosolic acid have been identified to transcriptionally up-regulate the expression of HO-1 [82,83].

Nrf2 is an essential transcription factor that coordinates the induction of genes encoding numerous cytoprotective enzymes such as NAD(P)H: quinone oxidoreductase-1 (NQO1), superoxide dismutase (SOD), glutathione S-transferase (GST), glutathione peroxidase (GPx), heme oxygenase-1 (HO-1), glutamate cysteine ligase (GCL), catalase, and thioredoxin [84,85]. It has been reported that Nrf2-deficient mice failed to induce genes responsible for protection against oxidative stress [86]. In normal condition, NRF2 resides in the cytoplasm as an inactive complex with the repressor known as Kelch-like ECH-associated protein 1 (Keap1) that functions as a sensor of cellular redox changes. When activated by conditions of oxidative stress, the structure of keap1 is altered and subsequently causes the release of Nrf2. Nrf2 is then translocated into the nucleus and dimerized with other transcription factors including small Maf (sMaf) forming a heterodimer and subsequently binds to antioxidant response elements (ARE) [84]. ARE is responsible of regulating the transcriptional of cytoprotective genes that are located in the promoter region of genes encoding various antioxidant and phase 2 detoxifying enzymes [84,87]. Therefore, it is likely that Keap-Nrf2-ARE pathway is be responsible for the increased induction of a number of antioxidant responses and phase II antioxidant genes by OPP. The activation of this pathway subsequently increases the cellular resistance to oxidative stress and leads to the protection against stress-related neurodegenerative disease development.

### 3.2. Anti-inflammatory Effects

The main cause of oxidative stress in neurodegenerative diseases is the inflammation caused by activated microglial cells that constitute up to 20% of the brain cell population [88]. Highly activated microglia but not astrocytes have been found in the substantia nigra of PD brains [89]. The activation of microglial cells has been associated with enhancement of signal transduction that subsequently leads to increased expression of inflammatory enzymes such as inducible nitric oxide synthase (iNOS) and cyclooxygenase-2 (COX-2), cytokines such as interleukin-1 (IL-1) and tumor necrosis factor-a (TNF-a) as well as transcription factors such as nuclear factor- κB (NF- κB) [90]. Postmortem brains from AD patients and from APP transgenic animals have shown an overexpression of inflammatory cytokines as well as chemokines including interferon γ (IFNγ), TNFα, interleukin 1β (IL-1β) and interleukin 6 (IL-6) [91]. Particularly, besides the induction of immunocytes migration across the blood-brain barrier as well as the engagement of astrocytes and endothelial cells, the activation of microglia affects neuronal viability through a persistent ROS and RNS generation. The inability to balance the production and elimination of ROS results in oxidative stress [92,93]. The oxidative stress-mediated inflammation has been associated with neurodegenerative disorders, including AD and PD [94,95]. The relevance of neuroinflammation in neurodegenerative disorders is derived from the evidence that it is detectable years before significant loss of neurons occurs [96].

Polyphenols have been reported to downregulate the pro-inflammatory cytokines such as iNOS and COX-2 via the suppression of Nuclear factor kappa-light-chain-enhancer of activated B cells (NF- κB) [97,98,99]. NF- κB is a family of inducible transcription factors, which regulate multiple aspects of different processes of the immune and inflammatory responses [100]. The activation of NF-κB occurs via two distinct kinase-dependent pathways: classical/canonical NF-κB pathway and alternative/non-canonical NF-κB pathway. The canonical NF-κB pathway is the most extensively studied, which can be mediated through various stimuli such as IL-1 receptor, Toll-like receptors (TLRs), and TNF receptor in response to pro-inflammatory mediators like IL-1, LPS, and TNF [101]. The NF-κB proteins are controlled by inhibitors of IκB family such as IκBα, IκBβ, IκBε, I κBγ, Bcl-3, p100 and p105 [102]. In its inactive state, NF-κB presents in the cytoplasm as a heterodimer comprising of p65 and p50, which bound to IκB subunits. The canonical NF-κB pathway which can be initiated via Toll-like receptor or cytokine receptor signaling depends on the inhibitor of κB kinase (I κK) complex that comprises of the kinases I κKα and I κKβ as well as the regulatory subunit I κKγ. Activated I κK complex phosphorylates the inhibitory subunit IκBα, triggering its degradation [101]. The released NF-κB (p50-p65) heterodimers are then translocated to the nucleus and subsequently bind κB site of chromosome, inducing the transcriptional activation of NF-κB-regulated genes including inflammatory cytokines (COX-2, iNOS, TNF-α), chemokines (RANTES, IP-10) and genes encoding adhesion molecules [101,102]. It has been reported that OPP had significantly reduced the protein levels of iNOS and COX-2 as well as inhibited NF- κB subunit p65 in a dose-responsive manner in the LPS-activated cells [55]. Moreover, OPP had significantly reduced the level of inflammatory chemokines such as IP-10, RANTES and TNFα in IL-1β-induced normal human astrocytes [56]. IP-10 is a member of the CXC chemokine family with pro-inflammatory that is involved in the recruitment of Th1 responses [103]. The Th1-type cytokines produce IL-2, IFN-γ and tumour necrosis factor-beta (TNF-β) that may contribute to the preservation of autoimmune reactions. An excessive pro-inflammatory reaction may subsequently cause abandoned tissue damage [104]. RANTES has been implicated as a mediator of neuronal injury that was found elevated in the brain of PD and AD model [105]. Furthermore, OPP has been observed to reduce the expression of cell adhesion molecules such as ICAM and VCAM in IL-1β-activated astrocytes. During inflammatory condition, circulating cell adhesion molecules are frequently found in the blood probably after being shed from the activated vascular and immune cells [106].

Therefore, as OPP are able to inhibit the NF-κB pathway, the pro-inflammatory response during neuroinflammation could also be halted. Meanwhile, the non-canonical NF-κB pathway might not be responsible for the anti-inflammatory actions of OPP since all the mediators involved are not downregulated by OPP. In brief, the non-canonical NF-κB pathway requires NF-κB inducing kinase (NIK) synthesis and is considered a slower kinetics compared to canonical pathway. When stimulated by specific stimuli such as LPS, lymphotoxin (LT) α1β2 and receptor activator of NF-κB (RANK), NIK is activated and recruits I κKα to the p100 complex to phosphorylate p100 leading to p100 ubiquitination. P52, the processing product of p100, generates the activated p52/RelB NF-κB complex, which can be translocated to the nucleus and induces the downstream gene expressions. Based on our readings, none of the pro-inflammatory molecules involved in non-canonical pathway have been downregulated by OPP [101].

**Figure 2:**
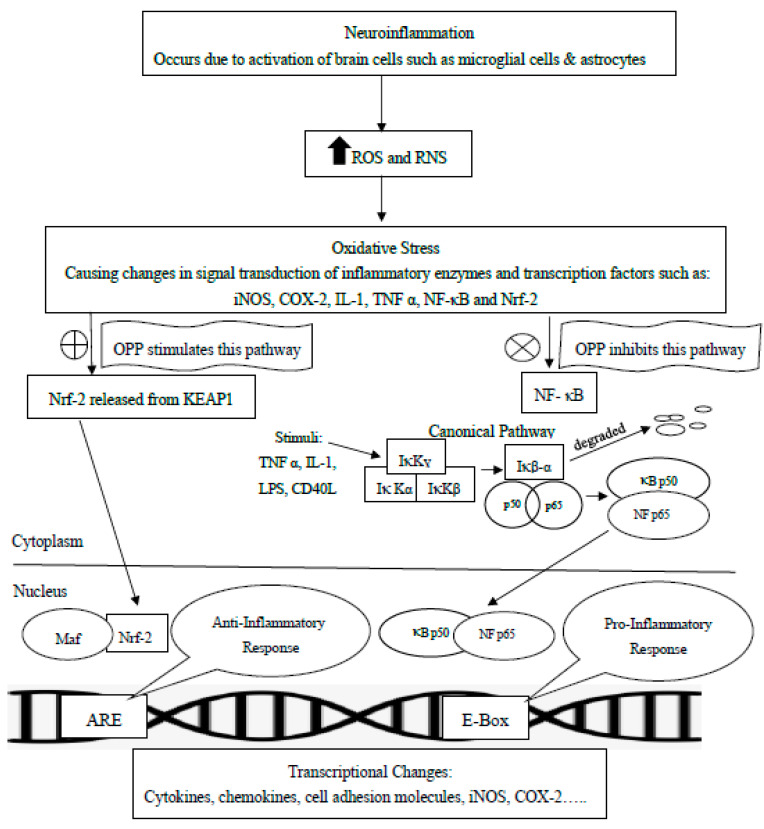
Canonical Pathway of NF-κB which represents the pro-inflammatory response, and Keap-Nrf2-ARE Pathway which represents anti-inflammatory response. Both pathways may potentially affected by OPP for attenuating neuroinflammation. ⊕: activate or stimulate; ⊗: inhibit; ARE: antioxidant response element; CD40L: CD 40 ligand; COX-2: cyclooxygenase-2; E-Box: Enhancer Box; iNOS: inducible nitric oxide synthase; IL-1: Interleukin-1; I κKα, β, ɣ: IκB Kinase α, β, ɣ; KEAP1: Kelch-like ECH-associated protein 1; LPS: Lipopolysaccharide; Maf: Maf proteins; NF- κB: Nuclear Factor kappa-light-chain-enhancer of activated B cells; Nrf2: nuclear factor erythroid 2-related factor 2; RNS: reactive nitrogen species; ROS: reactive oxygen species; TNF-α: Tumour Necrosis Factor Alpha. This figure is modified from Shih et al. [101] and Weinberg et al. [56].

Since OPP have been reported to significantly increase Nrf2 [55], this may suggest a reciprocal effect on NF-κB and Nrf2, following the consideration of homeostatic balance between the NF-κB pathway that usually activates pro-inflammatory genes and the Nrf2 pathway, which usually activates anti-inflammatory genes [96,107]. The reciprocal effects of these pro-inflammatory (NF-κB) and anti-inflammatory (Nrf2) are depicted as in Figure 2.

Anti-inflammatory effects of OPP have been also observed in the microarray assay performed by Leow et al. [42]. The researchers found that OPP was able to down-regulate genes involved with inflammatory process and genes in focal adhesion as well as alanine, aspartate, valine, leucine and isoleucine metabolisms. OPP has been observed to down-regulate Spp1 (secreted phosphoprotein 1 or osteopontin), Saa3 (serum amyloid A3) and Apod (apolipoprotein D), which are involved in inflammation [42]. Spp1 has been shown to be implicated in inflammatory and degenerative diseases of the nervous system.

For example, protein level of Spp1 was increased in multiple sclerosis during disease relapses [108]. Moreover, Spp1 has been considered as a potential biomarker in predicting the progression of mild cognitive impairment (MCI) to overt AD. This suggests that Spp1 is markedly increased in the common neurodegenerative disease; AD [109]. Therefore, any reduction in its level may indicate that the condition might have been improved. Serum amyloid A proteins play an important role in the immune system functions and persistent expression is implicated in the pathogenesis of chronic inflammatory diseases [110]. Meanwhile for ApoD, it has been identified as the most upregulated gene in the aged brain in a cross-species experiment involving human, macaque and mouse comparative analysis of transcription changes [111]. Besides aging, ApoD has been upregulated in a range of neurologic disorders including AD and other forms of dementia, Parkinson’s disease, motor neuron disease, bipolar disorder and schizophrenia. [112]. Therefore, the down-regulation of these three genes by OPP may indicate the ability of OPP as an anti-inflammatory agent to prevent brain aging and neurodegenerative development.

Other than that, the anti-inflammatory of OPP can be seen via its ability to inhibit Aβ42 aggregation, which is the primary component of amyloid plaque in the brains of Alzheimer’s patients. Alzheimer’s disease is associated with the aggregation of amyloid-β peptide (Aβ42/Aβ40) that develops into soluble neurotoxic oligomers, activating microglia and astrocytes to an acute inflammatory state that releases cytokines such as TNF-α, IL-1b, and IFN-γ. The Aβ peptide-induced neuroinflammatory state results in synaptic loss and neuronal death in the brain regions essential for cognitive function, causing the loss of memories, personalities and cognitive function resulting in dementia. The inhibitory effects by OPP may prevent the formation of neurotoxic oligomers and hold the potential to reduce neuroinflammation.

### 3.3. Modulation of Genes Regulated by Brain-Derived Neurotrophic Factor

BDNF is a part of neurotrophin family, which also consists of neural growth factor (NGF), neurotrophin 3 (NT3) and neurotrophin 4 (NT4). It has a wide array of functions within the brain and is highly present in several brain structures. Additionally, it also can be found in peripheral organs including the thymus, spleen, gut and heart. The major storage site for peripheral BDNF is platelet (90%) and only a small amount of free BDNF is present in plasma. In the brain, its functions include activity-dependent plasticity, neuronal survival, formation of new synapses, dendritic branching as well as the regulation of excitatory and inhibitory neurotransmitter profiles [113,114]. During brain pathological condition, a downregulation of BDNF release may occur, resulting in reduction of BDNF level in the brain and blood [115]. The reduced level of BDNF expression has been observed in many neurodegenerative diseases especially in Alzheimer’s disease [115] and Parkinson’s disease [116].

In the microarray gene expression analysis harvested from the brain of OPP-treated mice, there is an up-regulation of genes involved in neurotrophic activities such as calcium ion binding, calmodulin binding, potassium ion transport and transmembrane receptor protein tyrosine phosphatase activity. Besides, OPP also up-regulated genes involved in nervous system development, neurotransmitter transport, striated muscle contraction, synaptic transmission and synaptogenesis. Remarkably, majority of these genes are regulated by BDNF, which plays an important role in learning and memory. Previous studies have demonstrated the role of BDNF in learning and memory whereby increased BDNF mRNA were detected in hippocampus brain region, which is responsible for cognitive functions [117,118]. Particularly, the up-regulated genes regulated by BDNF were Arc, Cast or D14Ertd171e, Gria3, Kcnb1, Kcnab1, Homer1, Dlgap2, Dlgh4, Sv2b, Stx1a, Gucy1b3, Ncald, Bzrap1 and Pclo [42]. The up-regulation of genes in BDNF network by OPP may contribute to neuronal differentiation and survival as well as the maintenance of neuronal arborizations [119]. This may have subsequently caused improvement in memory and cognitive impairment [42].

Interestingly, it was found that OPP shares identical up-regulated neurotrophic genes with extracts of *Gingko biloba* leaves, which are a well-known dietary supplement with good efficiency to counteract depression, short-term memory loss as well as lack of attention. Those up-regulated genes are namely as tyrosine phosphatases (Ptprn and Ptprt) and ionotropic glutamate receptor (Gria3). Tyrosine phosphatase is important for the regulation of synapse dynamics and is closely associated with the breakdown of intracellular neurofibrillary tangles, an important feature of AD [120,121]. Meanwhile, ionotropic glutamate receptor (Gria3) mediates rapid excitatory neurotransmission contributing to synaptogenesis and neuronal circuitry for memory formation and learning processes [121].

Another gene up-regulated by OPP is Arc (activity-regulated cytoskeletal-associated protein), which is a master regulator of synaptic function that has a critical role in late-phase of long-term potentiation and memory consolidation [122]. The expression of Fos (Finkel–Biskis–Jinkins osteosarcoma) and Dlgh4 (discs large homolog 4) genes, which are the markers for neuronal and post-synaptic activities, are also up-regulated by OPP. The upregulation of Arc, Dlgh4 and Fos genes by OPP indicates that OPP may contribute to a well-regulated long-term synaptic function and induction of local dendritic targeted protein synthesis in the brain [42,119].

Apart from that, OPP may also improve the condition of neurodegenerative diseases via postsynaptic regulation. This is due to the ability of OPP to upregulate Dlgap2 and Homer1 gene expression, which are associated with postsynaptic density. Dlgap is a member of a family of Discs large associated proteins that function as scaffold proteins in the postsynaptic density (PSD). PSD is a highly specialized matrix that can be found in the synaptic terminal of postsynaptic neurons and participates in the neuronal signal transmission across the synaptic junction [123]. Meanwhile, homer proteins possess a role at the post-synaptic density as scaffolds, linking several molecules important for cellular signaling. Specifically, Homer1, has a vital function in synaptic plasticity and intracellular calcium signaling [124]. Therefore, via the upregulation of these genes, it can be stated that OPP are able to modulate synaptic signaling and prevent the neurodegenerative diseases’ development.

## 4. Conclusions and Future Perspectives

This review has summarized the direct effects and potential mechanisms of OPP on neuroprotective actions. Both in vitro and in vivo studies have shown promising results of this valuable nutraceutical derived from the oil palm fruit on neurodegenerative diseases, especially for Alzheimer’s disease. To our concern, apart from taking good supplementation to prevent and treat neurodegenerative diseases, people should remain mentally and socially active throughout their lives. For instance, people may opt to try new activities and taking part in group gatherings to obtain a healthy mental and social life.

For future direction, clinical trials should be performed to evaluate the applicability of these reported pre-clinical results and the proposed mechanisms of OPP in the human neuronal health. More studies using OPP on other important neurodegenerative diseases such as Parkinson’s disease and Huntington’s disease should be explored. In this review, the antioxidant and anti-inflammatory properties have been proposed as the responsible pathways for the neuroprotective actions of OPP. Besides, the modulation of genes involved in brain-derived neurotrophic factor is a potential mechanism for the therapeutic action of OPP on neuronal health. Further in-depth studies are warranted to explore a conclusive mechanism. The understanding of the proposed mechanism that leads to neuronal health will deliver more insight into the development of potent neuroprotective agents that specifically target the pathways. In conclusion, the direct effects of OPP related to neuronal health have been evidently revealed by literature with promising results in preventing and treating the neurodegenerative diseases.

## Figures and Tables

**Table 1:** The effects of OPP related to neuronal health.

Study Type	Sample/Population	Intervention Dose & Route	Findings	Reference
In vitro	Thioflavin T (ThT) binding assay	300 μL/10 mL of OPP concentration was added to Aβ 1–42 proteins.	ThT fluorescence intensity of the OPP treated assay was decreased compared to control.	[46]
	Familial AD and Wild-type B103 cells.	Both cells were incubated with OPP concentrations (150, 300, 500 μL/10 mL) for protein expression, while incubated with OPP concentration at 150, 300, 500 μL/10 mL for mRNA expression.	Protein expression levels APP In a dose-dependent manner, the APP protein expression in the FAD cells was decreased to the same level as the WT control. Additionally, OPP shows a minor reduction in APP levels even in the WT cells. COX-2 The addition of OPP had significantly decreased the amount of COX-2 protein expression to approximately the same level as the WT control. PARP The addition of OPP had significantly decrease the PARP protein expression in a dose dependent manner. mRNA levels APP There was no significant difference in APP mRNA level between the control and OPP-containing samples in FAD mutant cells. There was statistically significant fold difference in APP expression in WT cells when comparing WT control and WT 150 to WT 300 and WT 600 μL/10 mL samples. COX-2 In FAD cells, only 150 μL/10 mL OPP concentration showed significant difference in COX2 mRNA level while others did not show any changes. This might be related to contamination of the sample. For WT cells, its control and 150 μL/10 mL sample were statistically significant compared to 300 μL/10 mL sample and 600 μL/10 mL sample. In addition, the 300 μL/10 mL sample was statistically significant compared to 600 μL/10 mL sample.	
In vitro	Transgenic yeast assay	OPP at a concentration ranges (0–10 µg/mL) were added and incubated with the assay.	For Congo Red dye binding, there were 50% inhibition of Aβ aggregation (IC50) at an OPP concentration of 3.24 μg/mL. For the dynamic light scattering method, there were successive prolongation of the lag phase and a lower slope of exponential fibrillar growth phase at higher OPP concentration. For the mass spectrometry method, only the monomeric Aβ could be observed at concentrations of 90, 900,4500 and 9000 µg/mL OPP. For 2D-IR spectroscopy, beta-sheet signal was absent following 10 h of incubation at 10 µg/mL OPP. In the transgenic yeast assay, the presence of 10 μg/mL OPP rescues the growth of the β-amyloid-producing yeast, Huntingtin-producing yeast and TDP-43-producing yeast ranging from 40–190%.	[50]
In vitro	U87 MG glioma cells	OPP given at 800 µg/mL to the cell line	OPP supplementation had upregulated Phase II antioxidant genes such as PRDX2, HMOX1, XPA, GPX1, HSPB1, MAPK8, SOD1, PRdx1, GSTM4 and HIF1A in the cell line. OPP supplementation demonstrated a mild up-regulation of GSS and GCLC as well as increased the GSH/GSSG ratio of astrocytes in the cell line. OPP supplementation had upregulated HO-1 and nrf2 protein expression in the cell line.	[55]
In vitro	Liposaccharide (LPS) activated BV-2 cell line	OPP added at concentration 50 to 800 µg/mL	OPP had significantly reduced the protein levels of inflammatory enzymes such as iNOS, COX-2 and prostaglandin-2 in the LPS-activated cells. OPP treatment had also significantly reduced the protein levels of NF- κB in the LPS-activated cells.	[55]
In vitro	IL-1β-activated normal human astrocytes	OPP added at concentration ranges at 0, 0.9, 1.8, 3.6 mg/mL.	OPP had significantly reduced level of TNFα, RANTES, and IP-10 in a dose-dependent and time-dependent manner when compared to the control. OPP had significantly reduced ROS production by IL-1β-activated astrocytes. OPP reduced the expression of ICAM and VCAM, both in activated and non-activated human astrocytes in vitro.	[56]
In vivo	Male inbred BALB/c mice	OPP was given as drinking fluid for 6 weeks.	OPP treatment showed a downward trend in latency, mean distance and mean velocity in water maze trials when compared to control. OPP treatment showed an upward trend for average time, average distance travelled and average stopping speed before falling off the rotating drums in rotarod test compared to control. OPP have upregulated genes involved in brain development and activity such as Arc, Cast or D14Ertd171e, Gria3, Kcnb1, Kcnab1, Homer1, Dlgap2, Dlgh4, Sv2b, Stx1a, Gucy1b3, Ncald, Bzrap1 and Pclo. OPP had downregulated genes involved in inflammation such as Spp1, Saa3 and Apod. OPP had also downregulated genes involved in focal adhesion and metabolism of amino acid including alanine, aspartate, valine, leucine, and isoleucine.	[42]
In vivo	32 in-bred aged Brown Norway rats induced with high fat diet for 5 months (23 weeks)	OPP given as 5% purified-diet pellets	From month 1 to month 5, OPP- fed rats showed improvement in escape latency on Morris water maze test. OPP-treated rats revealed few traces of amyloid deposition compared to high-fat control rats in Congo red staining.	[53]
In vivo	32 in-bred aged Brown Norway rats induced with high fat diet for 5 months (23 weeks)	OPP given as 5% purified-diet pellets	OPP-fed rats had a significant improvement in Escape Latency of Morris Water Maze test (*p* < 0.05) compared to control and high-cholesterol groups OPP-treated group preserved more healthy neurons and demonstrated lower density of plaque deposition in the hippocampal CA-1 and DG areas when compared to high cholesterol fed diet rats in H&E staining. OPP had also significantly lower ß-amyloid 42 level when compared to control and high-cholesterol diet groups. OPP-fed group had also significantly lower plasma, liver and brain MDA level compared to high cholesterol fed group OPP-fed group had significantly lower 3-HOB level compared to high cholesterol group. OPP-fed group showed insignificant difference in IL-6 level compared to high cholesterol group OPP-fed group showed significantly lower gene expression of APP, BACE-1, and ApoE compared to high cholesterol diet group.	[43]

## References

[1] Gandhi S., Abramov A.Y. (2012). Mechanism of oxidative stress in neurodegeneration. Oxidative Med. Cell. Longev..

[2] Kim G.H., Kim J.E., Rhie S.J., Yoon S. (2015). The role of oxidative stress in neurodegenerative diseases. Exp. Neurobiol..

[3] Hou Y., Dan X., Babbar M., Wei Y., Hasselbalch S.G., Croteau D.L., Bohr V.A. (2019). Ageing as a risk factor for neurodegenerative disease. Nat. Rev. Neurol..

[4] Feigin V.L., Vos T. (2018). Global burden of neurological disorders: From global burden of disease estimates to actions. Neuroepidemiology.

[5] Brown R.C., Lockwood A.H., Sonawane B.R. (2005). Neurodegenerative diseases: An overview of environmental risk factors. Environ. Heal. Perspect..

[6] Hung C.-W., Chen Y.C., Hsieh W.-L., Chiou S.-H., Lao C.-L. (2010). Ageing and neurodegenerative diseases. Ageing Res. Rev..

[7] Pohl F., Lin P.K.T. (2018). The potential use of plant natural products and plant extracts with antioxidant properties for the prevention/treatment of neurodegenerative diseases: In Vitro, In Vivo and clinical trials. Moecules.

[8] Farrell S.R., Howlett S.E. (2008). The age-related decrease in catecholamine sensitivity is mediated by beta(1)-adrenergic receptors linked to a decrease in adenylate cyclase activity in ventricular myocytes from male Fischer 344 rats. Mech. Ageing Dev..

[9] Daubner S.C., Le T., Wang S. (2011). Tyrosine hydroxylase and regulation of dopamine synthesis. Arch. Biochem. Biophys..

[10] Bristow M.R., Ginsburg R., Minobe W., Cubicciotti R.S., Sagerman W.S., Lurie K., Billingham M.E., Harrison D.C., Stinson E.B. (1982). Decreased catecholamine sensitivity and beta-adrenergic-receptor density in failing human hearts. N. Engl. J. Med..

[11] Sahin E., Depinho R.A. (2010). Linking functional decline of telomeres, mitochondria and stem cells during ageing. Nat. Cell Biol..

[12] Chen X., Guo C., Kong J. (2012). Oxidative stress in neurodegenerative diseases. Neural Regen. Res..

[13] Liu Z., Zhou T., Ziegler A.C., Dimitrion P., Zuo L. (2017). Oxidative stress in neurodegenerative diseases: From molecular mechanisms to clinical applications. Oxidative Med. Cell. Longev..

[14] Zuo L., Zhou T., Pannell B.K., Ziegler A.C., Best T.M. (2015). Biological and physiological role of reactive oxygen species-The good, the bad and the ugly. Acta Physiol..

[15] Uttara B., Singh A.V., Zamboni P., Mahajan R.T. (2009). Oxidative Stress and Neurodegenerative Diseases: A Review of Upstream and Downstream Antioxidant Therapeutic Options. Curr. Neuropharmacol..

[16] Liu Z., Ren Z., Zhang J., Chuang C.-C., Kandaswamy E., Zhou T., Zuo L. (2018). Role of ROS and nutritional antioxidants in human diseases. Front. Physiol..

[17] Rafii M., Aisen P.S. (2009). Recent developments in Alzheimer’s disease therapeutics. BMC Med..

[18] Zandi P.P., Anthony J.C., Khachaturian A.S., Stone S.V., Gustafson D., Tschanz J.T., Norton M.C., Welsh-Bohmer K.A., Breitner J.C.S. (2004). Reduced risk of Alzheimer disease in users of antioxidant vitamin supplements. Arch. Neurol..

[19] Sambanthamurthi R., Tan Y., Sundram K., Abeywardena M., Sambandan T.G., Rha C., Sinskey A.J., Subramaniam K., Leow S.-S., Hayes K.C. (2011). Oil palm vegetation liquor: A new source of phenolic bioactives. Br. J. Nutr..

[20] Syarifah-Noratiqah S.-B., Zulfarina M.S., Ahmad S.U., Fairus S., Naina-Mohamed I. (2019). The pharmacological potential of Oil Palm Phenolics (OPP) individual components. Int. J. Med. Sci..

[21] Sambanthamurthi R., Rha C., Sinskey A., Tan Y.A., Wahid M.B. (2010). Oil palm phenolics as a source of shikimic acid-An MPOB and MIT collaboration. MPOB Inf. Ser..

[22] Bochkov D.V., Sysolyatin S.V., Kalashnikov A.I., Surmacheva I.A. (2012). Shikimic acid: Review of its analytical, isolation, and purification techniques from plant and microbial sources. J. Chem. Biol..

[23] Borah J. (2015). Shikimic acid: A highly prospective molecule in pharmaceutical industry. Curr. Sci..

[24] Soni M., Carabin I., Burdock G. (2005). Safety assessment of esters of p-hydroxybenzoic acid (parabens). Food Chem. Toxicol..

[25] Pejin B., Ciric A., Markovic J.D., Glamoclija J., Nikolic M., Petrović J. (2016). An insight into anti-biofilm and anti-quorum sensing activities of the selected anthocyanidins: The case study of Pseudomonas aeruginosa PAO1. Nat. Prod. Res..

[26] Pejin B., Ciric A., Markovic J.D., Glamoclija J., Nikolic M., Stanimirovic B., Sokovic M. (2015). Quercetin potently reduces biofilm formation of the strain Pseudomonas aeruginosa PAO1 in vitro. Curr. Pharm. Biotechnol..

[27] Peungvicha P., Temsiririrkkul R., Prasain J.K., Tezuka Y., Kadota S., Thirawarapan S.S., Watanabe H. (1998). 4-Hydroxybenzoic acid: A hypoglycemic constituent of aqueous extract of Pandanus odorus root. J. Ethnopharmacol..

[28] Peungvicha P., Thirawarapan S.S., Watanabe H. (1998). Possible mechanism of hypoglycemic effect of 4-hydroxybenzoic acid, a constituent of Pandanus odorus root. Jpn. J. Pharmacol..

[29] Scazzocchio B., Vari R., Filesi C., D’Archivio M., Santangelo C., Giovannini C., Iacovelli A., Silecchia G., Li Volti G., Galvano F. (2011). Cyanidin-3-O-β-glucoside and protocatechuic acid exert insulin-like effects by upregulating PPARγ activity in human omental adipocytes. Diabetes.

[30] Lin C.-Y., Huang C.-S., Huang C.-Y., Yin M.-C. (2009). Anticoagulatory, antiinflammatory, and antioxidative effects of protocatechuic acid in diabetic mice. J. Agric. Food Chem..

[31] Harini R., Pugalendi K.V. (2010). Antihyperglycemic effect of protocatechuic acid on streptozotocin-diabetic rats. J. Basic Clin. Physiol. Pharmacol..

[32] Wang D., Zou T., Yang Y., Yan X., Ling W. (2011). Cyanidin-3-O-β-glucoside with the aid of its metabolite protocatechuic acid, reduces monocyte infiltration in apolipoprotein E-deficient mice. Biochem. Pharmacol..

[33] Borate A.R., Suralkar A.A., Birje S.S., Malusare P.V., Bongale P.A. (2011). Antihyperlipidemic effect of protocatechuic acid in fructose induced hyperlipidemia in rats. Int. J. Pharma Bio Sci..

[34] Bouallagui Z., Han J., Isoda H., Sayadi S. (2011). Hydroxytyrosol rich extract from olive leaves modulates cell cycle progression in MCF-7 human breast cancer cells. Food Chem. Toxicol..

[35] Han J., Talorete T.P.N., Yamada P., Isoda H. (2009). Anti-proliferative and apoptotic effects of oleuropein and hydroxytyrosol on human breast cancer MCF-7 cells. Cytotechnology.

[36] FabianiMaria R., De Bartolomeo A., Rosignoli P., Servili M., Montedoro G.F., Morozzi G. (2002). Cancer chemoprevention by hydroxytyrosol isolated from virgin olive oil through G1 cell cycle arrest and apoptosis. Eur. J. Cancer Prev..

[37] Gonzalez-Correa J.A., Navas M.D., López-Villodres J.A., Trujillo M., Espartero J., De La Cruz J.P. (2008). Neuroprotective effect of hydroxytyrosol and hydroxytyrosol acetate in rat brain slices subjected to hypoxia–reoxygenation. Neurosci. Lett..

[38] Covas M.-I., Nyyssönen K., Poulsen H.E., Kaikkonen J., Zunft H.-J.F., Kiesewetter H., Gaddi A., De La Torre R., Mursu J., Bäumler H. (2006). The effect of polyphenols in olive oil on heart disease risk factors. Ann. Intern. Med..

[39] Schaffer S., Podstawa M., Visioli F., Bogani P., Müller A.W.E., Eckert G.P. (2007). Hydroxytyrosol-rich olive mill wastewater extract protects brain cells in vitro and ex vivo. J. Agric. Food Chem..

[40] Sambanthamurthi R., Tan Y., Sundram K., Hayes K.C., Abeywardena M., Leow S.-S., Sekaran S.D., Sambandan T.G., Rha C., Sinskey A.J. (2011). Positive outcomes of oil palm phenolics on degenerative diseases in animal models. Br. J. Nutr..

[41] Ibrahim N.I., Fairus S., Mohamed I.N. (2020). The effects and potential mechanism of oil palm phenolics in cardiovascular health: Ace. Nutrients.

[42] Leow S.-S., Sekaran S.D., Tan Y., Sundram K., Sambanthamurthi R. (2013). Oil palm phenolics confer neuroprotective effects involving cognitive and motor functions in mice. Nutr. Neurosci..

[43] Yan W. (2017). The in vivo effect of oil palm phenolics (opp) In Atherogenic Diet Induced Rats Model of Alzheimer’s Disease (ad). Nutrition and Food Science.

[44] Zhou Z.-Y., Tang Y.-P., Xiang J., Wua P., Jin H.-M., Wang Z., Mori M., Cai D. (2010). Neuroprotective effects of water-soluble Ganoderma lucidum polysaccharides on cerebral ischemic injury in rats. J. Ethnopharmacol..

[45] Nakajima Y., Shimazawa M., Mishima S., Hara H. (2009). Neuroprotective effects of Brazilian green propolis and its main constituents against oxygen-glucose deprivation Stress, with a gene-expression analysis. Phytotherapy Res..

[46] Michelle G.L. (2012). The Effects of Oil Palm Phenolics on Inflammation and Oxidative Stress in Relation to Amyloid Beta Plaques in Fad-Mutant and Wild Type B103 Cells. Master’s Thesis.

[47] Vorhees C.V., Williams M.T. (2006). Morris water maze: Procedures for assessing spatial and related forms of learning and memory. Nat. Protoc..

[48] Kao C.-F., Liu Y.-L., Yu Y.W.-Y., Yang A.C., Lin E., Ekuo P.-H., Tsai S.-J. (2018). Gene-based analysis of genes related to neurotrophic pathway suggests association of BDNF and VEGFA with antidepressant treatment-response in depressed patients. Sci. Rep..

[49] Giasson B.I., Lee V.M.-Y., Trojanowski J.Q. (2003). Interactions of amyloidogenic proteins. NeuroMolecular Med..

[50] Weinberg R., Koledova V.V., Shin H., Park J.H., Tan Y.A., Sinskey A.J., Sambanthamurhi R., Rha C. (2018). Oil palm phenolics inhibit the in vitro aggregation of β -amyloid peptide into oligomeric complexes. Int. J. Alzheimer’s Dis..

[51] Chen G.-F., Xu T.-H., Yan Y., Zhou Y.-R., Jiang Y., Melcher K., Xu H.E. (2017). Amyloid beta: Structure, biology and structure-based therapeutic development. Acta Pharmacol. Sin..

[52] Bourhim M., Kruzel M., Srikrishnan T., Nicotera T. (2007). Linear quantitation of Aβ aggregation using Thioflavin T: Reduction in fibril formation by colostrinin. J. Neurosci. Methods.

[53] Monplaisir K.M. (2016). Effect of Oil Palm Phenolics on Beta Amyloid Deposition in Cholesterol Induced Rat Model. of Alzheimer’s Disease: Histological Evidence. Master’s Thesis.

[54] Ullrich C., Pirchl M., Humpel C. (2010). Hypercholesterolemia in rats impairs the cholinergic system and leads to memory deficits. Mol. Cell. Neurosci..

[55] Sundaresan A.M. (2013). Oil Palm Phenolics Suppresses Oxidative Stress and Inflammation. Master’s Thesis.

[56] Weinberg R.P., Koledova V.V., Schneider K., Sambandan T.G., Grayson A., Zeidman G., Artamonova A., Sambanthamurthi R., Fairus S., Sinskey A.J. (2018). Palm fruit bioactives modulate human astrocyte activity in vitro altering the cytokine secretome reducing levels of TNFα, RANTES and IP-10. Sci. Rep..

[57] Villegas I., Sánchez-Fidalgo S., De La Lastra C.A. (2011). Chemopreventive effect of dietary curcumin on inflammation-induced colorectal carcinogenesis in mice. Mol. Nutr. Food Res..

[58] Iwatsubo T., Odaka A., Suzuki N., Mizusawa H., Nukina N., Ihara Y. (1994). Visualization of A beta 42(43) and A beta 40 in senile plaques with end-specific A beta monoclonals: Evidence that an initially deposited species is A beta 42(43). Neuron.

[59] Bedi K.C., Snyder N.W., Brandimarto J., Aziz M., Mesaros C., Worth A.J., Wang L.L., Javaheri A., Blair I.A., Margulies K.B. (2016). Evidence for intramyocardial disruption of lipid metabolism and increased myocardial ketone utilization in advanced human heart failure. Circulation.

[60] De Chaves E.P., Narayanaswami V. (2008). Apolipoprotein E and cholesterol in aging and disease in the brain. Future Lipidol..

[61] Chen Z., Jalabi W., Shpargel K.B., Farabaugh K.T., Dutta R., Yin X., Kidd G.J., Bergmann C.C., Stohlman S.A., Trapp B.D. (2012). Lipopolysaccharide-induced microglial activation and neuroprotection against experimental brain injury is independent of hematogenous TLR4. J. Neurosci..

[62] Mccord J.M. (2000). The evolution of free radicals and oxidative stress. Am. J. Med..

[63] Santos R.M., Lourenço C.F., Ledo A., Barbosa R.M., Rocha B.S. (2012). Nitric oxide inactivation mechanisms in the brain: Role in bioenergetics and neurodegeneration. Int. J. Cell Biol..

[64] Garthwaite J. (2008). Concepts of neural nitric oxide-mediated transmission. Eur. J. Neurosci..

[65] Steinert J.R., Chernova T., Forsythe I.D. (2010). Nitric oxide signaling in brain function, dysfunction, and dementia. Neuroscience.

[66] Poulsen H.E., Prieme H., Loft S. (1998). Role of oxidative DNA damage in cancer initiation and promotion. Eur. J. Cancer Prev..

[67] Fang Y.-Z., Yang S., Wu G. (2002). Free radicals, antioxidants, and nutrition. Nutrients.

[68] Kurutas E.B. (2015). The importance of antioxidants which play the role in cellular response against oxidative/nitrosative stress: Current state. Nutr. J..

[69] Hulbert A., Pamplona R., Buffenstein R., Buttemer W.A. (2007). Life and death: Metabolic rate, membrane composition, and life span of animals. Physiol. Rev..

[70] Aquilano K., Baldelli S., Rotilio G., Ciriolo M.R. (2008). Role of nitric oxide synthases in Parkinson’s disease: A review on the antioxidant and anti-inflammatory activity of polyphenols. Neurochem. Res..

[71] Mattson M.P., Magnus T. (2006). Ageing and neuronal vulnerability. Nat. Rev. Neurosci..

[72] Kasote D.M., Katyare S.S., Hegde M.V., Bae H. (2015). Significance of antioxidant potential of plants and its relevance to therapeutic applications. Int. J. Biol. Sci..

[73] Rice-Evans C.A., Miller N.J., Paganga G. (1996). Structure-antioxidant activity relationships of flavonoids and phenolic acids. Free Radic. Biol. Med..

[74] Halliwell B. (2001). Role of free radicals in the neurodegenerative diseases. Drugs Aging.

[75] Pomierny-Chamioło L., Moniczewski A., Wydra K., Suder A., Filip M. (2013). Oxidative stress biomarkers in some rat brain structures and peripheral organs underwent cocaine. Neurotox. Res..

[76] Owen A.D., Schapira A.H.V., Jenner P., Marsden C.D. (1996). Oxidative stress and Parkinson’s disease. Ann. N.Y. Acad. Sci..

[77] Ferrari C. (2000). Free radicals, lipid peroxidation and antioxidants in apoptosis: Implications in cancer, cardiovascular and neurological diseases. Biologia.

[78] Wu Y. (2015). The in vivo effect of oil palm phenolics (OPP) in atherogenic diet induced rat model of Alzheimer’s disease (AD). FASEB J..

[79] Grotto D., Maria L.S., Valentini J., Paniz C., Schmitt G., Garcia S.C., Pomblum V.J., Rocha J.B.T., Farina M. (2009). Importance of the lipid peroxidation biomarkers and methodological aspects FOR malondialdehyde quantification. Química Nova.

[80] Gozzelino R., Jeney V., Soares M.P. (2010). Mechanisms of cell protection by heme oxygenase-1. Annu. Rev. Pharmacol. Toxicol..

[81] Nitti M., Piras S., Brondolo L., Marinari U.M., Pronzato M.A., Furfaro A.L. (2018). Heme oxygenase 1 in the nervous system: Does it favor neuronal cell survival or induce neurodegeneration?. Int. J. Mol. Sci..

[82] Martin D., Rojo A.I., Salinas M., Diaz R., Gallardo G., Alam J., De Galarreta C.M.R., Cuadrado A. (2003). Regulation of heme oxygenase-1 expression through the phosphatidylinositol 3-kinase/akt pathway and the Nrf2 transcription factor in response to the antioxidant phytochemical carnosol. J. Biol. Chem..

[83] McNally S.J., Harrison E.M., Ross J.A., Garden O.J., Wigmore S.J. (2007). Curcumin induces heme oxygenase 1 through generation of reactive oxygen species, p38 activation and phosphatase inhibition. Int. J. Mol. Med..

[84] Surh Y.-J., Kundu J.K., Na H.-K. (2008). Nrf2 as a Master redox switch in turning on the cellular signaling involved in the induction of cytoprotective genes by some chemopreventive phytochemicals. Planta Medica.

[85] Eggler A.L., Gay K.A., Mesecar A. (2008). Molecular mechanisms of natural products in chemoprevention: Induction of cytoprotective enzymes by Nrf2. Mol. Nutr. Food Res..

[86] Chan K., Kan Y.W. (1999). Nrf2 is essential for protection against acute pulmonary injury in mice. Proc. Natl. Acad. Sci. USA.

[87] Raghunath A., Sundarraj K., Nagarajan R., Arfuso F., Bian J., Kumar A.P., Sethi G., Perumal E. (2018). Antioxidant response elements: Discovery, classes, regulation and potential applications. Redox Biol..

[88] Mosley R.L., Benner E.J., Kadiu I., Thomas M., Boska M.D., Hasan K., Laurie C., Gendelman H.E. (2006). Neuroinflammation, oxidative stress, and the pathogenesis of Parkinson’s disease. Clin. Neurosci. Res..

[89] Banati R.B., Daniel S.E., Blunt S.B. (1998). Glial pathology but absence of apoptotic nigral neurons in long-standing Parkinson’s disease. Mov. Disord..

[90] Mrak R.E., Griffin W.S.T. (2005). Glia and their cytokines in progression of neurodegeneration. Neurobiol. Aging.

[91] Hoozemans J.J., Veerhuis R., Rozemuller J.M., Eikelenboom P. (2006). Neuroinflammation and regeneration in the early stages of Alzheimer’s disease pathology. Int. J. Dev. Neurosci..

[92] Pizzino G., Irrera N., Cucinotta M., Pallio G., Mannino F., Arcoraci V., Squadrito F., Altavilla D., Bitto A. (2017). Oxidative stress: Harms and benefits for human health. Oxidative Med. Cell. Longev..

[93] Halliwell B. (1996). Antioxidants in human health and disease. Annu. Rev. Nutr..

[94] Esch T., Stefano G.B., Fricchione G.L., Benson H. (2002). Stress-related diseases—A potential role for nitric oxide. Med. Sci. Monit..

[95] McGeer P.L., McGeer E.G. (2004). Inflammation and neurodegeneration in Parkinson’s disease. Parkinsonism Rel. Disord..

[96] Hsieh H.-L., Yang C.-M. (2013). Role of redox signaling in neuroinflammation and neurodegenerative diseases. BioMed Res. Int..

[97] Hou C.W., Chen H.L., Tzen J.T.C., Jeng K.-C.G. (2003). Effect of sesame antioxidants on LPS-induced NO production by BV2 microglial cells. Neuroreport.

[98] Lau F.C., Joseph J.A., McDonald J.E., Kalt W. (2009). Attenuation of iNOS and COX2 by blueberry polyphenols is mediated through the suppression of NF-κB activation. J. Funct. Foods.

[99] Rasheed Z., Akhtar N., Anbazhagan A.N., Ramamurhy S., Shukla M., Haqqi T.M. (2009). Polyphenol-rich pomegranate fruit extract (POMx) suppresses PMACI-induced expression of pro-inflammatory cytokines by inhibiting the activation of MAP Kinases and NF-kappaB in human KU812 cells. J. Inflamm. (Lond).

[100] Liu T., Zhang L., Joo D., Sun S.-C. (2017). NF-κB signaling in inflammation. Signal. Transduct. Target. Ther..

[101] Shih R.-H., Wang C.-Y., Yang C.-M. (2015). NF-kappaB signaling pathways in neurological inflammation: A mini review. Front. Mol. Neurosci..

[102] Hoffmann A., Baltimore D. (2006). Circuitry of nuclear factor kappaB signaling. Immunol. Rev..

[103] Gotsch F., Romero R., Friel L., Kusanovic J.P., Espinoza J., Erez O., Than N.G., Mittal P., Edwin S., Yoon B.Y. (2007). CXCL10/IP-10: A missing link between inflammation and anti-angiogenesis in preeclampsia? The journal of maternal-fetal & neonatal medicine. J. Matern. -Fetal Neonatal Med..

[104] Viallard J.F., Pellegrin J.L., Ranchin V., Schaeverbeke T., Dehais J., Longy-Boursier M., Ragnaud J.M., Leng B., Moreau J.F. (1999). Th1 (IL-2, interferon-gamma (IFN-gamma)) and Th2 (IL-10, IL-4) cytokine production by peripheral blood mononuclear cells (PBMC) from patients with systemic lupus erythematosus (SLE). Clin. Exp. Immunol..

[105] Tripathy D., Thirumangalakudi L., Grammas P. (2010). RANTES upregulation in the Alzheimer’s disease brain: A possible neuroprotective role. Neurobiol. Aging.

[106] Ridet J., Privat A., Malhotra S., Gage F. (1997). Reactive astrocytes: Cellular and molecular cues to biological function. Trends Neurosci..

[107] Ramesh G., MacLean A.G., Philipp M.T. (2013). Cytokines and chemokines at the crossroads of neuroinflammation, neurodegeneration, and neuropathic pain. Mediat. Inflamm..

[108] Carecchio M., Comi C. (2011). The role of osteopontin in neurodegenerative diseases. J. Alzheimer’s Dis..

[109] Simonsen A.H., McGuire J., Hansson O., Zetterberg H., Podust V.N., Davies H.A., Waldemar G., Minthon L., Blennow K. (2007). Novel panel of cerebrospinal fluid biomarkers for the prediction of progression to Alzheimer dementia in patients with mild cognitive impairment. Arch. Neurol..

[110] Ray A., Ray B.K. (1999). Persistent expression of serum amyloid A during experimentally induced chronic inflammatory condition in rabbit involves differential activation of SAF, NF-kappa B, and C/EBP transcription factors. J. Immunol..

[111] Loerch P.M., Lu T., Dakin K.A., Vann J.M., Isaacs A., Geula C., Wang J., Pan Y., Gabuzda D.H., Li C. (2008). Evolution of the aging brain transcriptome and synaptic regulation. PLoS ONE.

[112] Thomas E.A., Sutcliffe J.G. (2002). The neurobiology of apolipoproteins in psychiatric disorders. Mol. Neurobiol..

[113] Edelmann E., Lessmann V., Brigadski T. (2014). Pre- and postsynaptic twists in BDNF secretion and action in synaptic plasticity. Neuropharmacology.

[114] Giacobbo B.L., Doorduin J., Klein H., Dierckx R.A.J.O., Bromberg E., De Vries E. (2018). Brain-derived neurotrophic factor in brain disorders: Focus on neuroinflammation. Mol. Neurobiol..

[115] Budni J., Bellettini-Santos T., Mina F., Garcez M.L., Zugno A.I. (2015). The involvement of BDNF, NGF and GDNF in aging and Alzheimer’s disease. Aging Dis..

[116] Wang Y., Liu H., Zhang B.-S., Soares J.C., Zhang X.Y. (2016). Low BDNF is associated with cognitive impairments in patients with Parkinson’s disease. Park. Relat. Disord..

[117] Mizuno M., Yamada K., Olariu A., Nawa H., Nabeshima T. (2000). Involvement of brain-derived neurotrophic factor in spatial memory formation and maintenance in a radial arm maze test in rats. J. Neurosci..

[118] Kesslak J.P., So V., CHoi J., Cotman C.W., Gomez-Pinilla F. (1998). Learning upregulates brain-derived neurotrophic factor messenger ribonucleic acid: A mechanism to facilitate encoding and circuit maintenance?. Behav. Neurosci..

[119] Yin Y., Edelman G.M., Vanderklish P.W. (2002). The brain-derived neurotrophic factor enhances synthesis of Arc in synaptoneurosomes. Proc. Natl. Acad. Sci. USA.

[120] Song G.J., Jung M., Kim J.-H., Park H., Rahman H., Zhang S., Zhang Z.-Y., Park D.H., Kook H., Lee I.-K. (2016). A novel role for protein tyrosine phosphatase 1B as a positive regulator of neuroinflammation. J. Neuroinflamm..

[121] Watanabe C.M.H., Wolffram S., Ader P., Rimbach G., Packer L., Maguire J.J., Schultz P.G., Gohil K. (2001). The in vivo neuromodulatory effects of the herbal medicine ginkgo biloba. Proc. Natl. Acad. Sci. USA.

[122] Leung H.-W., Foo G.W.Q., Vandongen A. (2019). Arc regulates transcription of genes for plasticity, excitability and Alzheimer’s disease. bioRxiv.

[123] Rasmussen A.H., Rasmussen H.B., Silahtaroglu A. (2017). The DLGAP family: Neuronal expression, function and role in brain disorders. Mol. Brain.

[124] Zhu J., Hafycz J., Keenan B.T., Guo X., Pack A., Naidoo N.N. (2020). Acute sleep loss upregulates the synaptic scaffolding protein, Homer1a, in non-canonical sleep/wake brain regions, claustrum, piriform and cingulate cortices. Front. Neurosci..

